# Tetrahydrocurcumin Attenuates NaIO_3_-Induced Retinal Oxidative Injury via Suppression of NOX2-Derived ROS-Mediated Apoptosis

**DOI:** 10.3390/antiox15060765

**Published:** 2026-06-18

**Authors:** Tzu-Chun Chen, Thuy-Lan-Thi Vo, Shang-Chun Tsou, Hui-Min David Wang, Inga Wang, Chen-Ju Chuang, Hui-Wen Lin, Yuan-Yen Chang

**Affiliations:** 1 Institute of Medicine, Chung Shan Medical University, Taichung 402, Taiwan; 2 Department of Microbiology and Immunology, School of Medicine, Chung Shan Medical University, Taichung 402, Taiwan; 3 Graduate Institute of Biomedical Engineering, National Chung Hsing University, Taichung 402, Taiwan; 4 Rehabilitation Sciences & Technology, University of Wisconsin-Milwaukee, Milwaukee, WI 53211, USA; 5 Emergency Department, St. Martin De Porres Hospital, Chiayi 600, Taiwan; 6 Department of Medical Research, Chung Shan Medical University Hospital, Taichung 402, Taiwan; 7 Clinical Laboratory, Chung Shan Medical University Hospital, Taichung 402, Taiwan

**Keywords:** tetrahydrocurcumin (THC), age-related macular degeneration (AMD), NaIO_3_, apoptosis, NOX2 and MEK/ERK signaling pathway

## Abstract

Oxidative stress is a major contributor to the development of age-related macular degeneration (AMD), and excessive oxidative stress can induce retinal pigment epithelium (RPE) dysfunction, apoptosis, and retinal degeneration. Nicotinamide adenine dinucleotide phosphate (NADPH) oxidase 2 (NOX2) is a major enzymatic source of reactive oxygen species (ROS); however, its mechanistic role in sodium iodate (NaIO_3_)-induced oxidative injury remains unclear. Tetrahydrocurcumin (THC), the major metabolite of curcumin, exhibits potent antioxidant and cytoprotective activities, but its protective effects against AMD-associated retinal degeneration have not been fully elucidated. In the present study, we investigated whether THC protects against NaIO_3_-induced ROS-mediated apoptosis in RPE cells through regulation of NOX2 signaling. *In vitro*, THC significantly attenuated NaIO_3_-induced cytotoxicity and prevented apoptosis by suppressing hydrogen peroxide (H_2_O_2_) production and intracellular ROS accumulation in ARPE-19 cells. THC also preserved mitochondrial membrane potential by inhibiting the Src/p47^phox^/NOX2 signaling pathway and subsequently attenuated mitochondria-mediated apoptotic signaling. Furthermore, THC markedly reduced the expression of apoptotic proteins, including Bax, cleaved caspase-3, and cleaved PARP, concomitantly with suppression of Ras/Raf/MEK/ERK signaling. Mechanistically, treatment with the selective NOX2 inhibitor GSK2795039 significantly attenuated NaIO_3_-induced ROS accumulation and mitochondrial depolarization, while co-treatment with THC further enhanced these protective effects. *In vivo*, THC ameliorated NaIO_3_-induced retinal structural abnormalities by preserving the outer nuclear layer (ONL), reducing caspase-3 expression, and improving pupillary light responses in mice. Collectively, these findings demonstrate that THC protects against NaIO_3_-induced retinal degeneration through suppressing NOX2-dependent oxidative stress and downstream Ras/Raf/MEK/ERK-mediated apoptotic signaling, highlighting its potential as a therapeutic candidate for AMD and other oxidative stress-related retinal disorders.

## 1. Introduction

Age-related macular degeneration (AMD) is the leading cause of vision loss in individuals over 55 and affects 6–9% of the population worldwide. The number of AMD cases is expected to rise from 196 million in 2020 to 288 million by 2040 [1,2,3]. The annual incidence ranges from 0.3 per 1000 individuals aged 55–59 to 36.7 per 1000 in those aged 90 and older [2]. AMD is a neurodegenerative, multifactorial retinal disease influenced by aging, smoking, high-fat diets, light exposure, alcohol, genetic factors, and environmental pollution, all of which also increase the risk of other eye conditions such as dry eye disease, glaucoma, and cataracts, negatively impacting quality of life and health outcomes [4,5].

Previous studies suggest that oxidative stress and inflammation play key roles in AMD etiology [6,7,8]. Although excessive reactive oxygen species (ROS) accumulation can induce retinal pigment epithelium (RPE) cell death, the precise mechanisms underlying RPE degeneration in AMD remain incompletely understood. Previous studies have demonstrated that multiple forms of programmed cell death, including apoptosis and dysregulated autophagy, contribute to RPE loss [9,10]. Nicotinamide adenine dinucleotide phosphate (NADPH) oxidase 2 (NOX2) is another major source of ROS in addition to mitochondria [11,12]. Notably, the NOX family is unique among enzyme systems in that its primary biological function is the generation of ROS, including superoxide anion radicals (O_2_^•−^) and hydrogen peroxide (H_2_O_2_). Studies have shown that NOX family members contribute to ROS overproduction, endoplasmic reticulum stress, autophagy, and apoptosis [13,14]. NOX2, also known as gp91^phox^, consists of membrane-bound NOX2 and p22^phox^ subunits and the cytoplasmic subunit p47^phox^ and the small GTPase Rac [15,16]. Under oxidative stress conditions, p47^phox^ translocates from the cytoplasm to the membrane and interacts with membrane-associated catalytic components, resulting in NOX2 activation [17]. Previous studies have shown that NOX2 expression is relatively low in normal retinal tissues and retinal endothelial cells, but is overexpressed in pathological retinal conditions such as ischemic or diabetic retinopathy [18]. Our previous studies demonstrated that NaIO_3_ induces ROS accumulation and activates caspase-dependent apoptotic signaling in RPE cells, ultimately contributing to retinal degeneration [7,19]. However, the mechanistic role of NOX2-derived oxidative stress in NaIO_3_-induced RPE injury has not yet been fully elucidated. To date, no studies have investigated whether tetrahydrocurcumin (THC) protects against NaIO_3_-induced retinal degeneration through modulation of NOX2-dependent oxidative stress signaling.

THC is the major metabolite of curcumin and exhibits relatively high stability under physiological conditions. Previous studies have demonstrated that THC possesses anti-inflammatory, hypoglycemic, hypolipidemic, and antioxidant activities [20,21]. THC has also been reported to provide neuroprotection against glutamate-induced neuronal cell death by inhibiting the accumulation of oxidative stress and phosphorylation of mitogen-activated protein kinases [MAPKs, including c-Jun N-terminal kinase (JNK), extracellular signal-regulated kinase (ERK), and p38] [22].

Interestingly, THC has been shown to attenuate obesity-induced skin damage by inhibiting NOX2, NOX4, and phosphorylated p65 expression, thereby reducing oxidative stress and inflammatory responses [23]. Furthermore, several studies have demonstrated that THC attenuates tissue damage by suppressing generation of ROS mediated through the MAPK pathway and NOX2 [24,25]. Therefore, the present study aimed to investigate the protective effects of THC against NaIO_3_-induced retinal degeneration and to determine whether THC attenuates NOX2-mediated ROS accumulation, mitochondrial dysfunction, and Ras/Raf/MEK/ERK-dependent apoptosis signaling in RPE cells.

## 2. Methods

### 2.1. Cell Culture

The ARPE-19 cell line (ATCC, Manassas, VA, USA) [8,26] was cultured in Dulbecco’s Modified Eagle Medium (DMEM; Cat# SH30022.02, GE Healthcare Life Sciences, HyClone Laboratories, Logan, UT, USA) supplemented with 10% heat-inactivated fetal bovine serum (FBS; Cat# 10437, Gibco, Thermo Fisher Scientific, Inc., Waltham, MA, USA), 100 μg/mL streptomycin, and 100 U/mL penicillin (Cat# 15140, Gibco, Waltham, MA, USA). Cells were maintained at 37 °C, 5% CO_2_, with medium changes every 2 days.

### 2.2. MTT Assay

ARPE-19 cells were seeded in 12-well plates at a density of 2 × 10^5^ cells/mL and pretreated with THC (0–50 µM) for 1.5 h. Subsequently, cells were incubated with or without NaIO_3_ (6 mM; Sigma-Aldrich, St. Louis, MO, USA) for 24 h. Cell viability was assessed using the MTT assay as previously described by [27], with MTT-formazan measured at 570–655 nm using an ELISA plate reader (Tecan Sunrise, Tecan Austria GmbH, Salzburg, Austria).

### 2.3. Intracellular ROS and H_2_O_2_ Level Analysis

After treatment, cells were incubated for 1 h with 2 μM H2DCF-DA. The dye was then removed, and cells were collected with 0.25% trypsin-EDTA. ROS levels were measured using a FACSCalibur flow cytometer (BD Biosciences, San Jose, CA, USA) measuring green fluorescence (FITC, 530/30 nm). Data analysis was performed with CellQuest software (version 3.1), and experimental conditions and procedures were performed as previously described by [6]. Statistical values were normalized to 100% based on the control group (mock) and presented as percentage values in a bar graph. The supernatant was used for the H_2_O_2_ assay using a commercial kit (ab102500, abcam, Milpitas, CA, USA) according to the manufacturer’s instructions. Absorbance was measured colorimetrically at 570 nm using an ELISA reader (Tecan Sunrise, Tecan Austria GmbH, Salzburg, Austria).

### 2.4. Mitochondrial Damage Assay

Mitochondrial damage was assessed using JC-1 staining (Cayman Chemical, Ann Arbor, MI, USA) and Hoechst 33342 (Invitrogen, Carlsbad, CA, USA), as described by [27].

### 2.5. Annexin V/PI Staining

To measure apoptosis levels, we applied Annexin V/propidium iodide dye (Annexin V-FITC Apoptosis Detection Kit, Enzo Life Sciences, Farmingdale, NY, USA) and imaged the samples using a FACSCalibur flow cytometer (BD Biosciences, CA, USA), following the method described by [6].

### 2.6. Western Blotting Assay

To assess the effects of THC on protein levels, Western blotting was performed using the following primary antibodies: cleaved-caspase-3 (sc-56053), Bax (sc-20067), Bcl-2 (sc-509), cleaved-PARP (sc-56196), K-RAS (sc-30), p-MEK1/2 (sc-81503), p-ERK1/2 (sc-31675), p47^phox^ (sc-14015), β-actin (sc-47778), GAPDH (sc-32233) (all from Santa Cruz Biotechnology, Dallas, TX, USA), p-Src (D4964) and p-c-Raf (56A6) from Cell signaling (Danvers, MA, USA), and NOX2 (DF6520, Affinity Biosciences, Cincinnati, OH, USA). Bands were visualized with an ECL detection kit (Top Bio Corp, New Taipei, Taiwan) and quantified using EvolutionCapt software (v18.02, Vilber Lourmat SAS, F-77601 Marne-la-Vallée Cedex 3, France).

### 2.7. Animal Model Experiment

All animal protocols were conducted in accordance with [19] and adhered to the Institutional Animal Care and Use Committee (IACUC) guidelines of Chung Shan Medical University (IACUC no. 2680). BALB/c mice (6–8 weeks old, 28–29 g) were obtained from the National Laboratory Animal Center, Taiwan, and housed under standard conditions with a 12:12 h light cycle at 22 ± 3 °C. Mice were divided into three groups (n = 6 per group) and fed until 8 weeks old. Experimental groups received the following treatments:

Mock group: PBS 1× (200 μL, i.p.) on day 1, followed 2 h later by PBS (200 μL, i.v.), and then PBS 1× (200 μL, i.p.) daily from days 2–7.

NaIO_3_ group: PBS 1× (200 μL, i.p.) on day 1, followed 2 h later by NaIO_3_ (40 mg/kg, i.v.), and then PBS 1× (200 μL, i.p.) daily from days 2–7.

NaIO_3_ + THC group: THC (25 mg/kg/day, i.p.) on day 1, followed 2 h later by NaIO_3_ (40 mg/kg, i.v.), and then THC (25 mg/kg/day, i.p.) daily from days 2–7.

Mice were euthanized on day 7 using isoflurane (No. 08547, Panion and BF Biotech Inc., Taipei, Taiwan), and eyeball samples were collected for analysis to evaluate the effects of THC and/or NaIO_3_.

### 2.8. Histopathology Assay

The effects of THC on retinal histology and imaging in mice were assessed as described in [6]. Mice eyeballs were collected, stained, and imaged. The entire retina, along with the inner nuclear layer (INL) and outer nuclear layer (ONL) thicknesses, was captured using an optical microscope (Olympus Optical, Tokyo, Japan). Measurements from six random areas were averaged for analysis. Cleaved-caspase-3 (clone 31A106, Santa Cruz Biotechnology, Santa Cruz, CA, USA) expression in the retina was evaluated using the BondMax automated staining system (Vision BioSystems Ltd., Newcastle Upon Tyne, UK).

### 2.9. Statistical Analysis

Data were presented as mean ± standard deviation (SD), with n ≥ 3. One-way ANOVA was used to assess differences in both *in vitro* and *in vivo* experiments, followed by Duncan’s post hoc test. A *p*-value < 0.05 was considered statistically significant. Different letters above the bars indicate significant differences between groups.

## 3. Results

### 3.1. THC Protects ARPE-19 Cells from NaIO_3_-Induced Damage

**Figure 1:**
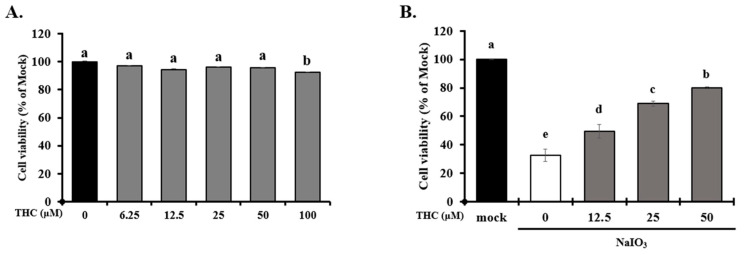
Effects of THC on cell survival in NaIO_3_-induced ARPE-19 cells. ARPE-19 cells were pretreated with THC (0–100 μM) for 1.5 h, and subsequently incubated in the absence (**A**) or presence (**B**) of 6 mM NaIO_3_ for 24 h. Cell viability was determined using the MTT assay, and absorbance was measured using a microplate reader. Data are presented as mean ± SD, n = 3, *p* < 0.05. Groups labeled with different letters (a–e) are significantly different from each other.

To evaluate the cytotoxicity of THC in ARPE-19 cells, cells were pretreated with THC (0–100 μM) for 1.5 h and co-incubated with or without 6 mM NaIO_3_ for 24 h. MTT assay results demonstrated that THC concentrations ≤50 μM did not significantly affect cell viability, whereas treatment with 100 μM THC markedly reduced cell survival, indicating concentration-dependent cytotoxicity at higher doses. Therefore, only non-cytotoxic concentrations (≤50 μM) were selected for subsequent experiments [Figure 1A, *p* < 0.05]. As shown in Figure 1B, NaIO_3_ treatment reduced cell viability to less than 40%, while THC pretreatment markedly improved cell survival in a dose-dependent manner, demonstrating its protective effects against NaIO_3_-induced retinal damage [Figure 1B, *p* < 0.05]. These findings indicate that THC effectively attenuates NaIO_3_-induced cytotoxicity in ARPE-19 cells under non-toxic experimental conditions.

### 3.2. Effects of THC on Src-p47^phox^-NOX2-Mediated ROS in NaIO_3_-Induced ARPE-19 Cells

NOX enzymes play a specific role in ROS generation, and their dysregulation or overexpression contributes to various degenerative and hyperproliferative pathological conditions. Among them, NOX2-derived ROS represents a major source of oxidative stress, and its excessive accumulation can induce retinal injury [28]. Recent studies have shown that NOX2 reduces ROS levels and stress-induced ferroptosis, thereby protecting retinal ganglion cells [29].

In diabetic retinopathy, hyperglycemia activates NOX2 through upregulation of RAC1 and p47^phox^, leading to increased cytoplasmic ROS production. This excessive ROS results in mitochondrial dysfunction and apoptosis, thereby contributing to disease progression [18].

**Figure 2:**
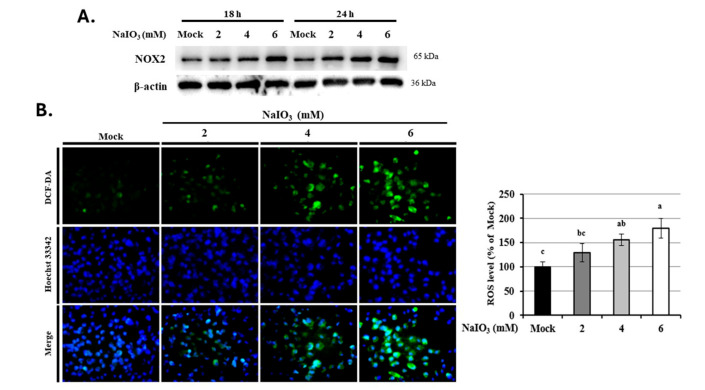

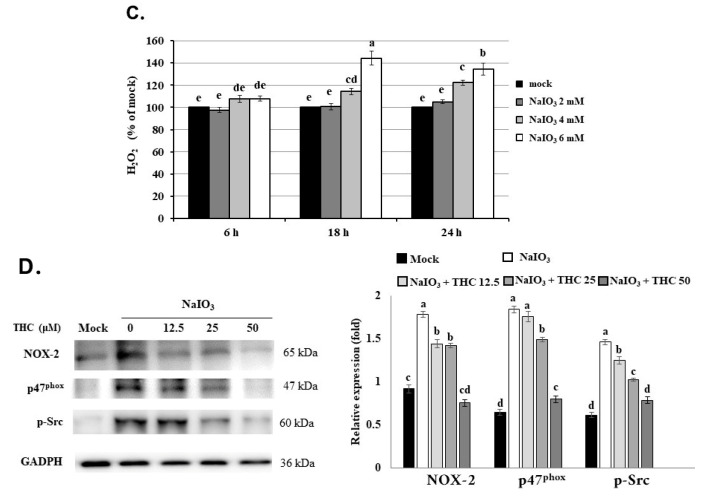
Effects of THC on Src-p47^phox^-NOX2-mediated ROS in NaIO_3_-induced ARPE-19 cells. ARPE-19 cells were pretreated without or with various concentrations of (0–50 μM) THC for 1.5 h, followed by incubation without or with different concentrations of NaIO_3_ (0, 2, 4, and 6 mM) for 18 or 24 h. (**A**) NOX2 protein expression analyzed by Western blot. (**B**,**C**) Intracellular ROS levels and cellular H_2_O_2_ production measured using flow cytometry and ELISA, respectively. (**D**) Western blot and quantitative analysis showing the effects of THC on NOX2 expression, as well as the effects of p47^phox^ and phosphorylation Src-family proteins, in NaIO_3_-treated ARPE-19 cells. Protein expression was quantified by densitometric analysis and normalized to β-actin or GAPDH as the loading control. Data are presented as mean ± SD, n ≥ 3, *p* < 0.05. Groups labeled with different letters (a–e) indicate statistically significant differences between them.

To determine whether NOX2 is involved in NaIO_3_-induced oxidative stress, we first examined NOX2 expression in ARPE-19 cells. As shown in Figure 2A, NOX2 expression increased in a dose-dependent manner when ARPE-19 cells were treated with different concentrations of NaIO_3_ (2, 4, and 6 mM) for 18 and 24 h. Consistently, intracellular ROS levels were significantly elevated in NaIO_3_-treated cells compared with untreated controls, also in a dose-dependent manner [Figure 2B]. Since ROS include both free radicals (such as superoxide and hydroxyl radicals) and non-radical species (such as H_2_O_2_), we further quantified H_2_O_2_ production using a commercial kit. As shown in Figure 2C, NaIO_3_ treatment significantly increased H_2_O_2_ levels in a concentration- and time-dependent manner (2, 4, and 6 mM for 18 or 24 h).

Previous studies have reported that Src plays an important role in the activation of NOX2 by inducing p47^phox^ expression in cell dysfunction [30,31]. To further investigate the regulatory role of NOX2 in NaIO_3_-induced oxidative stress, we evaluated the effect of THC. As shown in Figure 2D, NaIO_3_ exposure significantly increased NOX2 protein expression and induced the phosphorylation of its upstream signaling components, Src and p47^phox^. Conversely, THC pretreatment markedly suppressed NaIO_3_-induced NOX2 upregulation and inhibited the p47^phox^ and phosphorylation of Src. These results suggest that THC may inhibit NaIO_3_-induced ROS generation by suppressing NOX2 activation through the downregulation of the Src/p47^phox^ pathway.

### 3.3. THC Reduces NaIO_3_-Induced H_2_O_2_ Production and ROS Accumulation in ARPE-19 Cells

**Figure 3:**
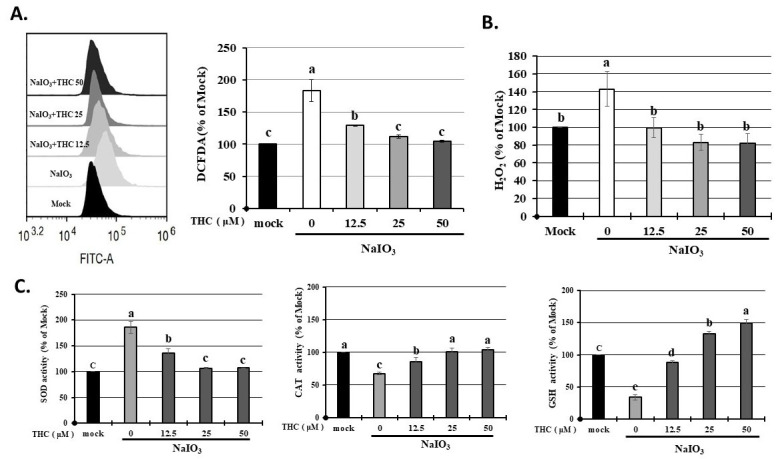
THC inhibits NaIO_3_-induced H_2_O_2_ generation and thus reduces ROS accumulation in ARPE-19 cells. ARPE-19 cells were first treated with different concentrations of THC (0–50 μM) for 1.5 h and then co-treated with NaIO_3_ for 18 h. (**A**) Intracellular ROS levels assessed by DCFDA staining and flow cytometry. (**B**) Cellular H_2_O_2_ production quantified by ELISA and (**C**) enzymatic activities of SOD and CAT, and intracellular levels of GSH in treated ARPE-19 cells, independently. Data are presented as the means ± SD of three independent experiments (n = 3). Different letters (a–e) in the statistical graphs indicate significant differences (*p* < 0.05) within each group.

Oxidative stress resulting from excessive ROS accumulation in RPE cells is recognized as a key pathological mechanism contributing to AMD development [32]. Excessive ROS accumulation in the RPE damages organelles and promotes the formation of extracellular macular sclerosis-like deposits, thereby causing RPE functional decline, cell apoptosis, or the induction of autophagy or disorders [6,7,33]. To investigate the effects of THC on NaIO_3_-induced oxidative stress, intracellular ROS levels were analyzed using DCFDA staining and flow cytometry, whereas H_2_O_2_ levels were measured using a commercial ELISA kit. As shown in Figure 3A, NaIO_3_ treatment significantly increased intracellular ROS levels compared with the control group. Pretreatment with THC markedly reduced ROS accumulation in a concentration-dependent manner, and treatment with 25 and 50 μM THC restored ROS levels close to those observed in untreated cells (*p* < 0.05). Consistently, NaIO_3_ exposure significantly increased H_2_O_2_ production, whereas THC pretreatment effectively suppressed H_2_O_2_ generation [Figure 3B].

To further determine whether THC modulates endogenous antioxidant defenses, the activities of antioxidant enzymes were examined. In physiological systems, cellular redox homeostasis is maintained through coordinated antioxidant systems, including superoxide dismutase (SOD), catalase (CAT), peroxidase (Prx), glutathione peroxidase (GPx), glutathione, vitamin C, and vitamin E [34]. As shown in Figure 3C, NaIO_3_ treatment significantly increased SOD activity while reducing CAT and GSH activities. THC pretreatment markedly attenuated the NaIO_3_-induced increase in SOD activity and restored CAT and GSH activities toward normal levels. Collectively, these findings indicate that THC effectively suppresses NaIO_3_-induced oxidative stress by reducing ROS and H_2_O_2_ accumulation and by restoring antioxidant defense capacity in ARPE-19 cells. These results indicate that the increased H_2_O_2_ expression in the NaIO_3_-induced group led to intracellular ROS accumulation, while THC pretreatment alleviated this phenomenon by inhibiting SOD activity and increasing CAT and GSH activities.

### 3.4. THC Attenuates NOX2-Dependent Oxidative Stress and Preserves Mitochondrial Function in NaIO_3_-Treated ARPE-19 Cells

Mitochondria play critical roles in cellular metabolism, biosynthesis, ion homeostasis, and redox regulation, and are closely associated with aging, apoptosis, immune responses, and cell survival [35]. Previous studies have suggested that NOX2-derived ROS production represents an early upstream event that precedes mitochondrial ROS accumulation and subsequent mitochondrial dysfunction [36]. In particular, Kowluru et al. demonstrated that inhibition of NOX2 effectively attenuated mitochondrial damage and capillary cell apoptosis under oxidative stress conditions [36]. Therefore, we further investigated whether THC could protect mitochondrial integrity against NaIO_3_-induced oxidative injury in ARPE-19 cells.

**Figure 4:**
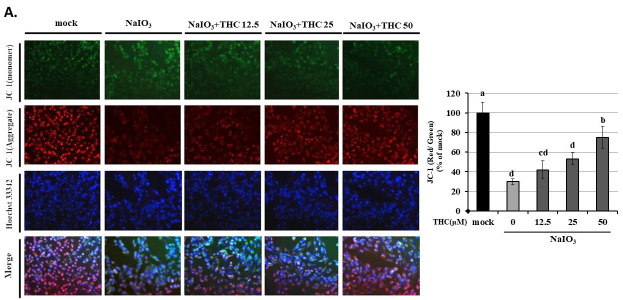

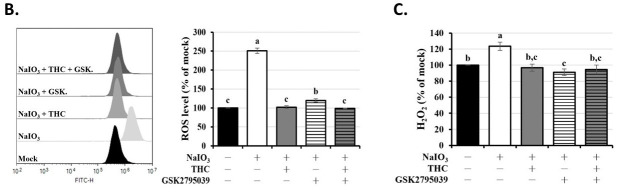

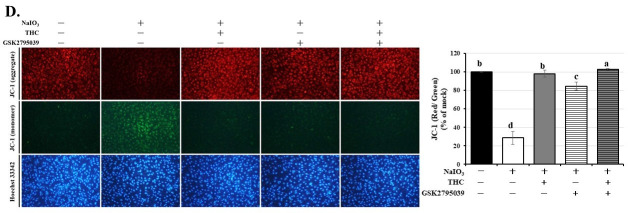
THC attenuates NOX2-dependent oxidative stress and mitochondrial dysfunction in NaIO_3_-treated ARPE-19 cells. (**A**) ARPE-19 cells were pretreated with THC (0–50 μM) for 1.5 h and subsequently exposed to NaIO_3_ for 18 h. Mitochondrial membrane potential (ΔΨm) was assessed by JC-1 staining. Red fluorescence indicates intact mitochondria, whereas green fluorescence indicates mitochondrial depolarization. (**B**) Intracellular ROS levels were measured by DCFDA staining and flow cytometry. (**C**) Extracellular H_2_O_2_ production was quantified using an ELISA-based assay. (**D**) Quantitative analysis of mitochondrial membrane potential based on the JC-1 red/green fluorescence ratio. For mechanistic studies (**B**–**D**), ARPE-19 cells were pretreated with GSK2795039 (10 μM), THC (50 μM), or their combination for 1.5 h prior to NaIO_3_ exposure. Data are presented as the means ± SD of three independent experiments (n = 3). Different letters (a–d) in the statistical graphs indicate significant differences (*p* < 0.05) within each group.

To evaluate mitochondrial membrane potential (ΔΨm), JC-1 staining was performed following NaIO_3_ treatment. As shown in Figure 4A, exposure to NaIO_3_ for 18 h markedly increased the accumulation of JC-1 monomers (green fluorescence), indicating mitochondrial depolarization and loss of mitochondrial function. In contrast, THC pretreatment significantly promoted the formation of JC-1 aggregates (red fluorescence), indicative of preserved mitochondrial membrane integrity, and this protective effect was enhanced in a dose-dependent manner. These findings suggest that THC effectively preserves mitochondrial function and protects against NaIO_3_-induced oxidative mitochondrial injury in ARPE-19 cells.

To further clarify whether NaIO_3_-induced oxidative stress is mediated through NOX2 activation, we conducted additional mechanistic experiments using GSK2795039, a highly selective NOX2 inhibitor. ARPE-19 cells were pretreated with GSK2795039 alone or in combination with THC prior to NaIO_3_ exposure, followed by analysis of intracellular ROS levels, H_2_O_2_ production, and mitochondrial membrane potential. Intracellular ROS generation was quantified using DCFDA fluorescence staining and flow cytometric analysis, whereas extracellular H_2_O_2_ levels were measured using an ELISA-based assay. As shown in Figure 4B, NaIO_3_ treatment significantly increased intracellular ROS accumulation compared with the control group. Pretreatment with GSK2795039 markedly attenuated ROS production, while co-treatment with THC further suppressed ROS accumulation, providing direct pharmacological evidence that NOX2-derived ROS serves as a major upstream source of oxidative stress following NaIO_3_ exposure. Similarly, NaIO_3_ exposure significantly elevated H_2_O_2_ levels, whereas GSK2795039 pretreatment effectively reduced H_2_O_2_ production, and combined treatment with THC further enhanced this inhibitory effect (Figure 4C).

To determine whether NOX2-mediated oxidative stress contributes to mitochondrial dysfunction, mitochondrial membrane potential was further quantified using JC-1 fluorescence ratio analysis. As shown in Figure 4D, NaIO_3_ treatment significantly reduced the red/green fluorescence ratio, indicating severe mitochondrial depolarization. Pretreatment with THC significantly restored mitochondrial membrane potential, whereas GSK2795039 alone also markedly attenuated mitochondrial dysfunction. Notably, combined treatment with GSK2795039 and THC exhibited superior protective efficacy in stabilizing mitochondrial membrane potential compared with GSK2795039 alone. Collectively, these findings suggest that NOX2-derived oxidative stress is a major upstream contributor to NaIO_3_-induced mitochondrial dysfunction and that THC protects mitochondrial integrity, at least in part, through suppression of NOX2-dependent ROS generation and subsequent mitochondrial dysfunction.

### 3.5. THC Attenuates NaIO_3_-Induced Apoptosis and Apoptosis-Related Protein Expression in ARPE-19 Cells

**Figure 5:**
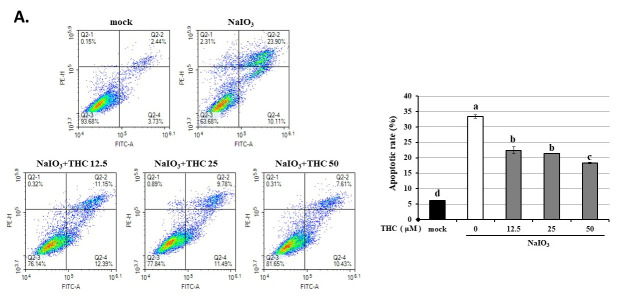

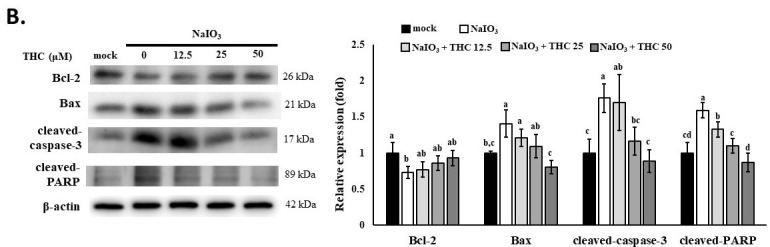
THC attenuates NaIO_3_-induced apoptosis and expression of apoptosis-related proteins in ARPE-19 cells. The ARPE-19 cells were pretreated with different concentrations of THC (0–50 μM) for 1.5 h and then co-treated with NaIO_3_ for 24 h. (**A**) Cell apoptosis was analyzed by Annexin V-FITC/propidium iodide (PI) staining and flow cytometry. (**B**) Expression levels of apoptosis-related proteins (Bcl-2, Bax, cleavage caspase 3, and cleavage PARP). Protein expression was quantified by densitometric analysis and normalized to β-actin as the loading control. Data are presented as the means ± SD of three independent experiments (n = 3). Different letters (a–d) in the statistical graphs indicate significant differences (*p* < 0.05) within each group.

Previous studies have demonstrated that NaIO_3_ induces apoptosis in ARPE-19 cells [6,7,19]. Therefore, we further investigated the mechanism by which THC alleviated NaIO_3_-induced cell death. Annexin V-FITC/PI dual staining followed by flow cytometry was performed to quantify apoptotic cells, and Western blot analysis was employed to examine the expression of key apoptosis-related factors. As shown in Figure 5A, NaIO_3_ treatment markedly induced apoptosis, elevating the apoptotic rate to approximately 34% of cells, compared to 7% in the mock group. THC pretreatment significantly attenuated this effect, reducing the apoptotic rate to around 23% in both the 12.5 and 25 μM THC-treated groups, with no significant difference observed between them. Notably, 50 μM THC further reduced the apoptotic population to 17% (*p* < 0.05).

As shown in Figure 5B, NaIO_3_ treatment markedly increased the expression of apoptosis-related proteins such as cleaved-caspase-3, Bax, and cleaved-PARP compared with the control group. In contrast, pretreatment with THC significantly attenuated the expression of these apoptosis-related proteins. In terms of the performance of anti-apoptotic proteins, it was also found that NaIO_3_ reduced Bcl-2 expression, while THC pretreatment restored Bcl-2 levels. These findings indicate that THC protects ARPE-19 cells against NaIO_3_-induced apoptosis by suppressing pro-apoptotic signaling and preserving anti-apoptotic protein expression.

### 3.6. THC Suppresses NaIO_3_-Induced Activation of the Ras/Raf/MEK/ERK Signaling Pathway in ARPE-19 Cells

**Figure 6:**
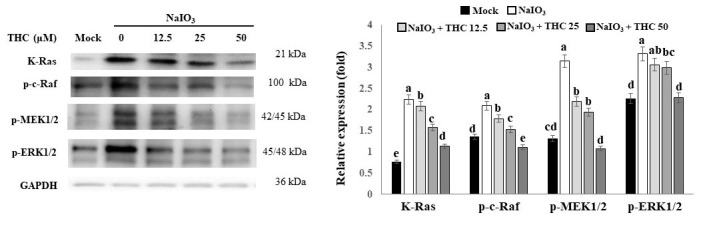
THC suppresses NaIO_3_-induced activation of the Ras/Raf/MEK/ERK signaling pathway. The ARPE-19 cells were pretreated with different concentrations of THC (0–50 μM) for 1.5 h and then co-treated with NaIO_3_ for 24 h. The protein levels of Ras, Raf, p-MEK, and p-ERK in ARPE-19 cells were analyzed by Western blotting. Protein expression was calculated from densitometry absorbance values of three separate experiments after they were corrected for GAPDH expression to obtain equal loading. Data are presented as the means ± SD of three independent experiments (n = 3). Different letters (a–e) in the statistical graphs indicate significant differences (*p* < 0.05) within each group.

Recent studies have shown NOX2 activation through Src/extracellular signal-regulated kinase (ERK)-dependent pathway in neurodegeneration [37]. Studies have also shown that oxidized low-density lipoprotein activates NOX2 through the MAPK pathway to produce ROS, thereby inducing the formation of neutrophil extracellular traps [38]. Furthermore, Lee et al. reported that H_2_O_2_-mediated ERK1/2 activation is on Ras and Raf signaling, and can subsequently induce apoptotic cell death in mouse L929 cells [39]. These findings suggest that oxidative stress may act upstream of the Ras/Raf/MEK/ERK signaling cascade. Therefore, we further investigated whether activation of the Ras/Raf/MEK/ERK pathway contributes to the apoptosis in NaIO_3_-treated ARPE-19 cells and whether THC can modulate this signaling cascade. As shown in Figure 6, NaIO_3_ treatment significantly increased the expression of Ras and the phosphorylation levels of c-Raf, MEK1/2, and ERK1/2 compared with the control group. In contrast, pretreatment with THC markedly suppressed the activation of these signaling molecules in a dose-dependent manner. These findings indicate that THC attenuates NaIO_3_-induced activation of the Ras/Raf/MEK/ERK pathway, which is associated with reduced oxidative stress, preservation of mitochondrial function, and suppression of apoptotic signaling in ARPE-19 cells.

### 3.7. THC Attenuates NaIO_3_-Induced Retinal Degeneration and Functional Impairment in Mice

**Figure 7:**
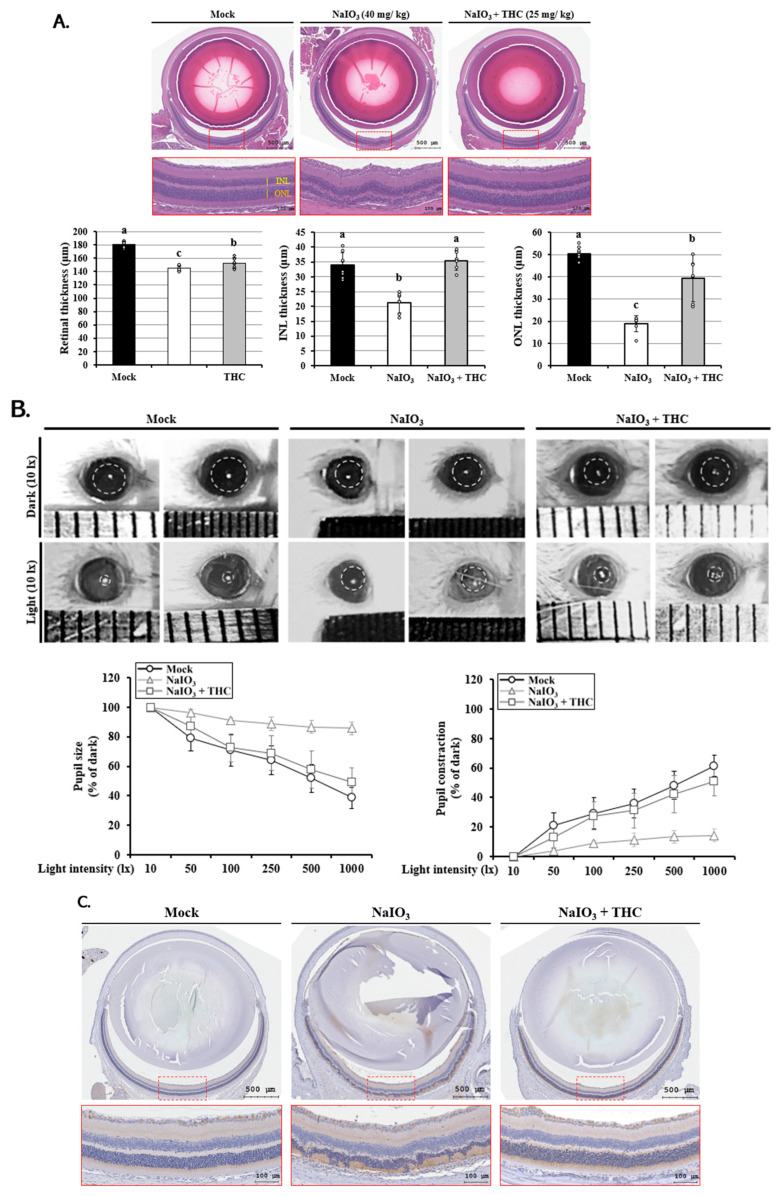
THC attenuates NaIO_3_-induced retinal degeneration and functional impairment in mice. (**A**) H&E staining of retinas from the mock, NaIO_3_-treated, and THC pretreatment + NaIO_3_ groups. Quantitative analysis of total retinal, inner nuclear layer (INL), and outer nuclear layer (ONL) thickness is shown (n = 6 mice/group). (**B**) Assessment of retinal function by pupillary light reflex (PLR) analysis. Representative images and quantitative analysis of pupil constriction responses following light stimulation are shown for the mock, NaIO_3_-treated, and THC + NaIO_3_ groups (n = 6 mice/group). (**C**) Immunohistochemical staining of apoptosis marker caspase 3 in retina tissues from the mock, NaIO_3_-treated, and THC + NaIO_3_ groups (n = 6 mice/group). Data are presented as the means ± SD of six independent experiments (n = 6). Different letters (a–c) in the statistical graphs indicate significant differences (*p* < 0.05) within each group.

To determine whether THC protects against NaIO_3_-induced retinal injury *in vivo*, retinal morphology, visual function, and apoptotic protein expression were evaluated in mice following daily THC administration. As shown in Figure 7A, hematoxylin and eosin (H&E) staining revealed that NaIO_3_ treatment markedly disrupted retinal architecture, as evidenced by thinning of the outer nuclear layer (ONL), inner nuclear layer (INL), and photoreceptor inner/outer segments (ISs/OSs). In addition, accumulation of migratory cells and cellular debris, together with loss of normal retinal layer organization, was observed in NaIO_3_-treated mice. In contrast, THC administration significantly attenuated these histopathological alterations and preserved retinal structural integrity.

To further evaluate retinal function, pupillary light reflex (PLR) analysis was performed [27,40]. As shown in Figure 7B, pupils from all experimental groups exhibited normal dilation under dark-adapted conditions. Following light stimulation, mice in the control group displayed rapid pupillary constriction, whereas NaIO_3_-treated mice showed a markedly impaired constriction response, indicating retinal dysfunction. Notably, THC treatment partially restored pupillary constriction compared with the NaIO_3_-treated group. Quantitative analysis further demonstrated that the percentage of pupil size after light exposure was significantly greater in the NaIO_3_ group than in the control group, whereas THC treatment significantly improved this response, indicating preservation of retinal function.

Previous research results have also confirmed that NaIO_3_ can induce apoptosis in RPE cells, so we also used an immunohistochemistry stain to detect caspase-3, a key marker of apoptosis. As shown in Figure 7C, NaIO_3_ treatment markedly increased cleaved caspase-3 expression (indicated by red arrows) compared to the mock group. However, THC administration significantly reduced cleaved caspase-3 expression in retinal tissues. Taken together, these results suggest that THC attenuates NaIO_3_-induced retinal degeneration, preserves retinal function, and suppresses apoptosis *in vivo*.

## 4. Discussion

Retinal degeneration is generally regarded as a progressive and irreversible process driven by complex interactions between genetic susceptibility and environmental factors, which ultimately lead to RPE cell death and vision loss. Among them, AMD is the most common cause of retinal degeneration and can be classified into two major forms: dry AMD, characterized by RPE cell degeneration, and wet AMD, characterized by choroidal neovascularization [5]. In addition to genetic predisposition, environmental factors such as oxidative stress, inflammatory response, and hypoxia are also believed to be closely associated with retinal degeneration. In recent years, studies have shown that excessive accumulation of ROS within RPE cells plays a central role in the development and progression of dry AMD by promoting cellular dysfunction, mitochondrial damage, and cell death [33].

Oxidative stress leads to RPE cell death and is widely used as a disease model to recapitulate pathological processes associated with AMD. Consequently, NaIO_3_-induced oxidative injury has been extensively used as an experimental model to mimic key pathological features of retinal degeneration and dry AMD [41,42]. In this study, we carefully selected THC concentrations to minimize inherent cytotoxicity while maintaining sufficient biological activity to assess its protective mechanisms under oxidative stress. The results showed that THC concentrations ≤ 50 μM had no significant effect on ARPE-19 cell viability, while 100 μM THC induced significant cytotoxicity, demonstrating a concentration-dependent biphasic effect. Therefore, subsequent experiments used only non-cytotoxic concentrations to ensure that the observed protective effects were not interfered with by direct toxic responses. Notably, THC pretreatment significantly restored cell viability after NaIO_3_ treatment, accompanied by reduced ROS accumulation, maintenance of mitochondrial membrane potential, and inhibition of apoptosis signaling. These findings indicate that the selected THC treatment conditions provide a biologically responsive and therapeutically relevant window for studying oxidative stress-mediated retinal protection, without confounding effects from nonspecific toxicity.

Previous studies have demonstrated that members of the NADPH oxidase (NOX) family are important enzymatic sources of ROS and contribute to retinal injury in various ocular diseases, including choroidal neovascularization, oxygen-induced retinopathy, and diabetic retinopathy [43,44,45]. In human retinal endothelial cells, all known NOX isoforms, including NOX1–5 and DUOX1/2, have been identified and shown to participate in the regulation of retinal vascular homeostasis through ROS generation [44]. Under pathological conditions, dysregulated NOX activity results in excessive ROS production, leading to oxidative damage, mitochondrial dysfunction, and retinal neurodegeneration. However, the specific contribution of NOX2-mediated oxidative stress to NaIO_3_-induced RPE injury has not been fully elucidated.

Our previous studies have shown that oxidative stress plays a key role in AMD, and NaIO_3_ induces oxidative stress, apoptosis, and autophagy in RPE cells, leading to retinal dysfunction [7,19,46]. In the present study, NaIO_3_ treatment markedly increased NOX2 expression, accompanied by elevated intracellular ROS accumulation and H_2_O_2_ production, ultimately resulting in ARPE-19 cell death (Figure 1 and Figure 2). At the molecular level, we observed that exposure to NaIO_3_ induced the phosphorylation of Src and p47^phox^, alongside the upregulation of NOX2 protein expression; however, pretreatment with THC significantly reduced the expression and activation of these proteins (Figure 2D). Furthermore, THC inhibited the production of H_2_O_2_ (Figure 3B) and subsequent excess ROS, thereby enhancing the cellular antioxidant defense capacity through the restoration of CAT and GSH activities (Figure 2 and Figure 3).

To further clarify the precise mechanistic role of NOX2-mediated oxidative stress in mitochondrial dysfunction, we additionally employed GSK2795039, a highly selective NOX2 inhibitor, in combination with THC treatment. Consistent with previous reports demonstrating that NOX2-derived ROS acts upstream of mitochondrial oxidative injury, pharmacological inhibition of NOX2 by GSK2795039 significantly attenuated NaIO_3_-induced intracellular ROS accumulation and H_2_O_2_ production, while simultaneously preserving mitochondrial membrane potential (Figure 4B–D). Notably, combined treatment with GSK2795039 and THC conferred greater protection than GSK2795039 alone, suggesting that THC-mediated retinal protection involves the suppression of NOX2-dependent oxidative stress in addition to complementary, parallel antioxidant mechanisms. These findings provide direct functional evidence supporting the concept that NOX2-derived ROS is a major upstream contributor to mitochondrial depolarization and subsequent apoptotic signaling during NaIO_3_-induced retinal injury.

Ras signaling is known to play many cellular functions and can regulate cell proliferation, apoptosis, migration, fate determination, and differentiation by regulating downstream Raf kinase [47]. Accumulating evidence suggests that oxidative stress-mediated mitochondrial dysfunction is closely associated with activation of the Ras/Raf/MEK/ERK signaling cascade in retinal degeneration models. Previous studies demonstrated that H_2_O_2_-mediated ERK1/2 activation is Ras- and Raf-dependent and can directly induce apoptotic cell death [48], supporting the concept that oxidative stress acts upstream of the Ras/Raf/MEK/ERK apoptotic signaling pathway. In addition, previous retinal oxidative stress studies have shown that NaIO_3_-induced apoptosis in ARPE-19 cells is mediated through activation of the MEK/ERK pathway, and pretreatment with selective MEK/ERK inhibitors, including U0126 and PD98059, significantly attenuated ROS-mediated apoptosis and mitochondrial dysfunction [6,46]. Furthermore, studies have demonstrated that curcumin-related compounds can modulate the Ras/Raf/MEK/ERK cascade under oxidative stress conditions [49].

Consistent with these previous findings, our results demonstrated that NaIO_3_ exposure significantly increased the phosphorylation of Ras, Raf, MEK1/2, and ERK1/2, whereas THC pretreatment markedly suppressed the activation of this signaling cascade (Figure 6). Importantly, inhibition of Ras/Raf/MEK/ERK activation by THC occurred concomitantly with the suppression of ROS accumulation, preservation of mitochondrial membrane potential (Figure 4), and reduction of apoptotic signaling.

**Figure 8:**
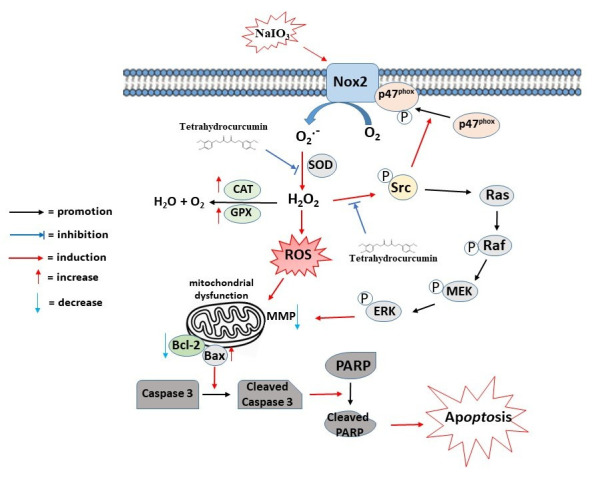
Proposed mechanism underlying the protective effects of tetrahydrocurcumin (THC) against NaIO_3_-induced oxidative damage in retinal pigment epithelial cells. THC suppresses NaIO_3_-induced NOX2 activation and ROS generation through inhibition of the Src/p47^phox^/NOX2 signaling pathway, thereby reducing oxidative stress and H_2_O_2_ accumulation. Consequently, THC preserves mitochondrial membrane potential, attenuates mitochondrial dysfunction, and suppresses apoptosis-associated protein activation, including Bax, cleaved caspase-3, and cleaved PARP. In addition, THC inhibits activation of the Ras/Raf/MEK/ERK signaling cascade, ultimately protecting ARPE-19 cells from oxidative stress-induced apoptosis and retinal degeneration.

To further delineate the hierarchical relationship between the pathways evaluated in this study, our findings strongly suggest that the Src/p47^phox^/NOX2 axis and the Ras/Raf/MEK/ERK cascade operate together within a unified upstream-downstream signaling network rather than as independent events. Specifically, NaIO_3_ exposure drives the initial activation of the Src/p47^phox^/NOX2 complex, culminating in a profound burst of intracellular ROS and H_2_O_2_ (Figure 2 and Figure 3). These elevated ROS levels then act as potent secondary messengers that cross-talk with and stimulate the redox-sensitive Ras/Raf/MEK/ERK pathway, a progression that is well-supported by our finding that pharmacological NOX2 inhibition via GSK2795039 effectively dampens downstream mitochondrial depolarization. By suppressing NOX2 activation, THC successfully truncates this signaling cascade at its inception, preventing the subsequent downstream activation of Ras/Raf/MEK/ERK and ultimately preserving RPE cell viability (as schematically summarized in Figure 8).

It is worth noting that while both autophagy and apoptosis are intricate components of the cellular stress response in RPE degeneration, their precise roles often depend on the intensity and duration of the oxidative stimulus. Accumulating evidence from numerous scholars has previously demonstrated that autophagy typically functions as an early, adaptive, pro-survival mechanism aimed at mitigating mild oxidative damage through the clearance of damaged organelles [50]. However, under severe, acute, or sustained chemical insult—such as the specific dosage of NaIO_3_ employed in this study—this protective autophagic capacity is frequently overwhelmed or its autophagic flux disrupted, eventually triggering a molecular cross-talk that shifts the cellular equilibrium toward programmed cell death [51,52].

Because our phenotypic characterization and Western blot analyses consistently identified a robust and predominant induction of classic, terminal apoptotic markers (including increased Annexin V-positive populations, Bax upregulation, and the distinct cleavage of caspase-3 and PARP), we focused our mechanistic elucidation primarily on the apoptotic cascade. This allows us to directly trace how THC effectively rescues ARPE-19 cells from terminal oxidative execution without confounding systemic variables. Consequently, THC successfully attenuated the expression of pro-apoptotic proteins (such as Bax, the cleaved form of caspase-3, and cleaved-PARP) while restoring the levels of the anti-apoptotic protein Bcl-2 in NaIO_3_-induced ARPE-19 cells (Figure 5B). These findings suggest that THC-mediated retinal protection is at least partially associated with the suppression of oxidative stress-dependent Ras/Raf/MEK/ERK activation and subsequent mitochondrial apoptotic signaling.

In this study, a NaIO_3_-induced mouse model was employed to determine whether THC could protect against retinal degeneration *in vivo*. Consistent with previous reports, NaIO_3_ treatment induced marked retinal structural alterations, including photoreceptor IS/OS loss, and ONL thinning and disorganization, indicating successful establishment of a retinal degeneration model [6,46,49]. Notably, daily administration of THC significantly attenuated these pathological changes, suggesting a protective effect against NaIO_3_-induced retinal injury (Figure 7A). This protective effect may be associated with the suppression of apoptosis, as THC markedly reduced cleaved caspase-3 expression in retinal tissues compared with the NaIO_3_-treated group (Figure 7C). This was confirmed by pupillary response testing of mice; THC indeed restored normal pupillary responses in NaIO_3_-treated mice (Figure 7B).

In conclusion, this study demonstrates that THC protects against NaIO_3_-induced retinal degeneration both *in vitro* and *in vivo*. Mechanistically, THC attenuated oxidative stress, preserved mitochondrial function, suppressed apoptotic signaling, and improved retinal structure and function. These protective effects were associated with inhibition of NOX2-mediated ROS generation and suppression of the Ras/Raf/MEK/ERK signaling pathway, highlighting THC as a promising therapeutic candidate for oxidative stress-related retinal disorders (Figure 8).

## 5. Conclusions

In summary, the present study demonstrates that THC exerts powerful cytoprotective and histological rescue effects against NaIO_3_-induced retinal degeneration across both *in vitro* and *in vivo* experimental models. Mechanistically, our findings successfully map a unified, hierarchical signaling network driving RPE cell death: NaIO_3_ exposure initiates an upstream oxidative cascade governed by the activation of the Src/p47^phox^/NOX2 complex, and the resulting robust burst of intracellular ROS and H_2_O_2_ acts as a critical secondary messenger axis that cross-talks with and drives the downstream, redox-sensitive Ras/Raf/MEK/ERK pro-apoptotic pathway.

Crucially, pharmacological intervention with THC successfully intercepts this degenerative cascade at its inception by suppressing upstream NOX2 activation, which in turn mitigates severe oxidative injury, preserves mitochondrial membrane potential, and prevents downstream apoptotic execution. Furthermore, our *in vivo* findings translate these molecular mechanisms into physiological outcomes, confirming that daily systemic administration of THC effectively mitigates outer retinal structural atrophy, maintains outer nuclear layer integrity, and preserves visual reflex function as verified by pupillary light responses. Taken together, these convergent lines of evidence validate the targeted therapeutic efficacy of THC, highlighting its clinical potential as a promising pharmacological candidate for the prevention and treatment of dry age-related macular degeneration (dry AMD) and other oxidative stress-driven geographic retinopathies.

## Data Availability

The original contributions presented in this study are included in the article. Further inquiries can be directed to the corresponding authors.
